# Roles of the miR-139-5p/CCT5 axis in hepatocellular carcinoma: a bioinformatic analysis

**DOI:** 10.7150/ijms.57504

**Published:** 2021-08-25

**Authors:** Jingjing Xu, Yuan Zhang, Cheng Liu, Ping Yan, Zongguo Yang

**Affiliations:** 1 Department of Pathology, Shanghai Public Health Clinical Center, Fudan University, Shanghai 201508, China.; 2 Department of Integrative Medicine, Shanghai Public Health Clinical Center, Fudan University, Shanghai 201508, China.; 3 Department of Infectious Disease, Putuo Hospital, Shanghai University of Traditional Chinese Medicine, Shanghai 200062, China.

**Keywords:** miR-139-5p, Chaperonin containing TCP1 subunit 5, CCT5, hepatocellular carcinoma, survival

## Abstract

**Background:** MiRNAs are pivotal regulators involved in proliferation, apoptosis, invasion, metastasis, epithelial-mesenchymal transition (EMT), angiogenesis, drug resistance and autophagy in hepatocellular carcinoma (HCC). The aim of this study was to investigate the influence of miR-139-5p and its target genes on the outcomes of HCC.

**Methods:** Survival analysis of miR-139-5p in HCC was conducted in Kaplan-Meier plotter. Target genes of miR-139-5p were identified in TargetScan, miRTarBase and starBase. Gene Expression Omnibus (GEO) series were used for the validation of miR-139-5p target genes. Cox proportional regression model was also established.

**Results:** In Kaplan-Meier plotter, 163 HCC patients were included. MiR-139-5p downregulation was significantly associated with unfavorable overall survival (OS) and disease-free survival (DFS) in HCC patients (all *P* < 0.001). MiR-139-5p was significantly downregulated in HCC tumors and human hepatoma cell lines (all *P* < 0.05). As a target gene of miR-139-5p, CCT5 was overexpressed in HCC tumor tissues and peripheral blood mononuclear cells (all *P* < 0.05). A negative correlation between CCT5 and miR-139-5p was found in TCGA dataset. CCT5 overexpression was significantly associated with worse OS in HCC patients (*P* < 0.001), which was validated in the GSE14520 dataset (*P* = 0.017). CCT5 mRNA was significantly overexpressed in HCC patients with alpha-fetoprotein (AFP) > 300 ng/ml, BCLC staging B-C, TNM staging III and main tumor size > 5 cm (all *P* < 0.05). According to the Cox regression model of CCT5-interacting genes, HCC patients with high risk had poor OS compared to those with low risk in the TCGA dataset (*P* < 0.001), with the 1-year, 3-year, and 5-year ROC curves of an area under the curve (AUC) equal to 0.704, 0.662, and 0.631, respectively.

**Conclusions:** MiR-139-5p suppresses HCC tumor aggression and conversely correlated with CCT5. The miR-139-5p/CCT5 axis might perform crucial functions in the development of HCC.

## Introduction

As a class of noncoding, highly conserved endogenous RNAs of approximately 18~25 nucleotides in length, microRNAs (miRNAs) can bind to specific mRNA sites and regulate mRNA expression for cleavage or translational repression 1, 2. Substantial evidence has revealed that miRNAs participate in various vital biological processes 3. Aberrant miRNA expression is implicated in numerous disorders, especially cancer. The important roles of miRNAs in malignant biological behavior display their potential as promising diagnostic and prognostic biomarkers 4.

As reviewed by Xu et al, miRNAs are pivotal regulators involved in proliferation, apoptosis, invasion, metastasis, epithelial-mesenchymal transition (EMT), angiogenesis, drug resistance and autophagy in hepatocellular carcinoma (HCC). Unfortunately, this group did not discuss the roles of miR-139 in the development of HCC 5. As a recognized tumor suppressor, miR-139-5p has been widely investigated in many malignancies, including breast cancer^6^, gastric cancer 7, colon cancer 8, bladder cancer 9, 10, lung cancer 11, prostate cancer 12, glioma 13, ovarian cancer 14, endometrial cancer 15, leukemia 16, 17, osteosarcoma 18, 19, esophageal cancer 20, 21 and oral squamous carcinoma 22. Conversely, a report by Pang C et al showed that miR-139-5p expression was significantly upregulated in prostate cancer patients compared with benign prostatic hyperplasia patients and healthy individuals 23. A bioinformatic analysis illustrated that miR-139-5p promotes the aggressiveness of adrenocortical cancer 24.

The interference of miR-139-5p could reverse the inhibitory effects of sevoflurane treatment on breast cancer cell migration, invasion, and EMT 25, and reverse the EMT of colon cancer stem cells 26. The miR-139-5p impeded the EMT in pancreatic cells^27^, cervical cancer cells 28, glioma 13, osteosarcoma 19, colorectal cancer cells 29, and in post-menopausal women with interstitial cystitis 30. In HCC, miR-139-5p functions to inhibit EMT and metastasis by targeting zinc finger E‑box binding to homeobox 1 (ZEB1) and ZEB2, and its expression is downregulated in HCC cells and tumor tissues 31, 32. MiR-139-5p inhibited HCC cell viability, migration and invasion and induced apoptosis by targeting kazal‐like domains proteoglycan 1 (SPOCK1), E26 transformation-specific 1 (ETS1) and NTRK like family member 4 (SLITRK4) 32-34. Given the controversial role of miR-139-5p in human malignancies and the importance of identifying target genes of miR-139-5p in HCC, we conducted an integrated bioinformatic analysis to investigate the roles and target genes of miR-139-5p in HCC patients.

## Materials and methods

### Data source

Survival analysis of miR-139-5p and its potential target genes was conducted in the Kaplan Meier plotter platform 35, 36. This database is processed by a PostgreSQL server, which integrates both gene expression and clinical data. To analyze the prognostic value of a particular gene, patient samples were divided into two groups by the proposed median cutoff of the biomarkers. Two patient groups were compared by Kaplan-Meier survival plot and the hazard ratio (HR) with 95% confidence intervals (CI) and log rank *P* value were calculated. When the miRNA ID hsa-miR-139-5p and non-commercial spotted platform were selected, 166 HCC patients were included in the analysis. Outcomes including overall survival (OS) and disease-free survival (DFS) were considered. No sex, tumor stage, cirrhosis status, metastasis, HBV infection, alcohol use or race restrictions.

Total RNA from 14 pairs of HCC tumors and adjacent tissues was isolated and purified in the GSE84402 dataset 22 for expression analysis of target genes of miR-139-5p. The GSE14520 dataset 37, 38 was used to validate relationships between miR-139-5p target genes and the survival and clinico-pathological characteristics of HCC patients. Data extraction methods used for the GSE14520 dataset have been described previously 39, 40.

### Cell culture

The normal liver cells and hepatoma cell lines (HLF, HCCLM3, Hep3B, Huh6, and Huh7) were obtained from the American Type Culture Collection (ATCC, MD, USA). Cells were cultured in Dulbecco's modified Eagles' medium (DMEM/high glucose) supplemented with 10% fetal bovine serum (FBS) (Cellsera, NSW, Australia) containing 50 µg/ml penicillin and 50 µg/ml streptomycin at 37 °C in a 5% CO2 incubator. The medium was changed three times every week 41.

### Quantitative Real time-PCR (qRT-PCR)

TaKaRa MiniBEST Universal RNA Extraction Kit (TaKaRa, Japan) was applied to extract the total RNA from samples according to the manufacture's recommendation. The reverse transcription of 500 ng total RNA was utilizing PrimeScript™ RT reagent Kit (TaKaRa, Japan) and quantitative real-time PCR with TB Green® Premix Ex Taq™ II (TaKaRa, Japan). The PCR was set at the initial denaturation of 2 min at 95 °C, following with 5 s at 95 °C, and 30 s at 60 °C in a total of 40 cycles, and was set at melt curve stage of 15 s at 95 °C, 1 min at 60 °C and 15s at 95 °C. All experiments were carried out in triplicate. The miR-139-5p expression was normalized to U6. The relative expression ratios of miR-139-5p were computed by the 2-ΔΔCT method. The primers involved in this assay were shown as follows: hsa-miR-139-5p, F: ACACTCCAGCTGGGTCTACAGTGCACGTG, R: GTGCAGGGTCCGAGGT; U6, F: AGAGAAGATTAGCATGGCCCCTG, R: ATCCAGTGCAGGGTCCGAGG.

### MiR-139-5p target gene prediction

The TargetScan v7.2 42, miRTarBase 43, 44 and starBase v3.0 45, 46 databases were used for miR-139-5p target gene prediction. In both the TargetScan v7.2 and miRTarBase databases, human species were selected, and miR-139-5p was entered into the microRNA name dialog box. In the starBase database, the miRNA-target intersections function was used, and the following criteria were selected: human genome, hg19 assembly, miR-139-5p, CLIP-data ≥ 1, degradome-data ≥ 0, and program number ≥ 1. Common target genes of miR-139-5p in these three databases were calculated and drawn by the Venn diagram webtool (http://bioinformatics.psb.ugent.be/webtools/Venn/). The expression levels between tumor and nontumor tissues, including gene expression in peripheral blood mononuclear cells (PBMCs) between healthy individuals and HCC patients, were validated in the Gene Expression Omnibus (GEO) series with Raw.CEL files. Gene expression was calculated with the Affy 47 and Limma 48 packages in the R program. We have previously reported the detailed microarray processing approaches of gene expression in these GEO series 39.

### Target gene expression and protein-protein-interaction analysis

The intersection of target genes of miR-139-5p and differentially expressed genes (DEGs) in the GSE84402 dataset from the GEO database was identified with the Venn diagram webtool (http://bioinformatics.psb.ugent.be/webtools/Venn/). Protein-protein-interaction analysis of miR-139-5p target genes of Homo sapiens organism was performed with the STRING v11.0 database (https://string-db.org/). Full network interacted proteins with interaction score more than 0.4 were selected.

### Cox proportional hazard regression model establish

A Cox proportional hazard regression model was established based on The Cancer Genome Atlas (TCGA) database using the R program. The edgeR package 49 was used to identify DEGs in HCC tumor and adjacent tissues, the survival package 50 was used to conduct univariate and multivariate Cox regression analyses, and then the Cox regression formula was calculated. According to this formula, HCC patients were divided into two groups, the high- and low-risk groups 51. Survival analysis between these two groups was also performed by the survival package 50. The timeROC package was used to generate a receiver operating characteristic (ROC) curve of the model for the prediction of OS in HCC patients.

### Statistical analysis

GraphPad Prism v7.0 (GraphPad Software, CA, US) was used. Student's *t* test or the Mann-Whitney *U* test were performed to analyze the differences in gene expression the two groups. Kaplan-Meier survival analysis was performed on the GSE14520 dataset. A two-tailed *P* < 0.05 was considered significant.

## Results

### Association between miR-139-5p and HCC survival

**Figure 1:**
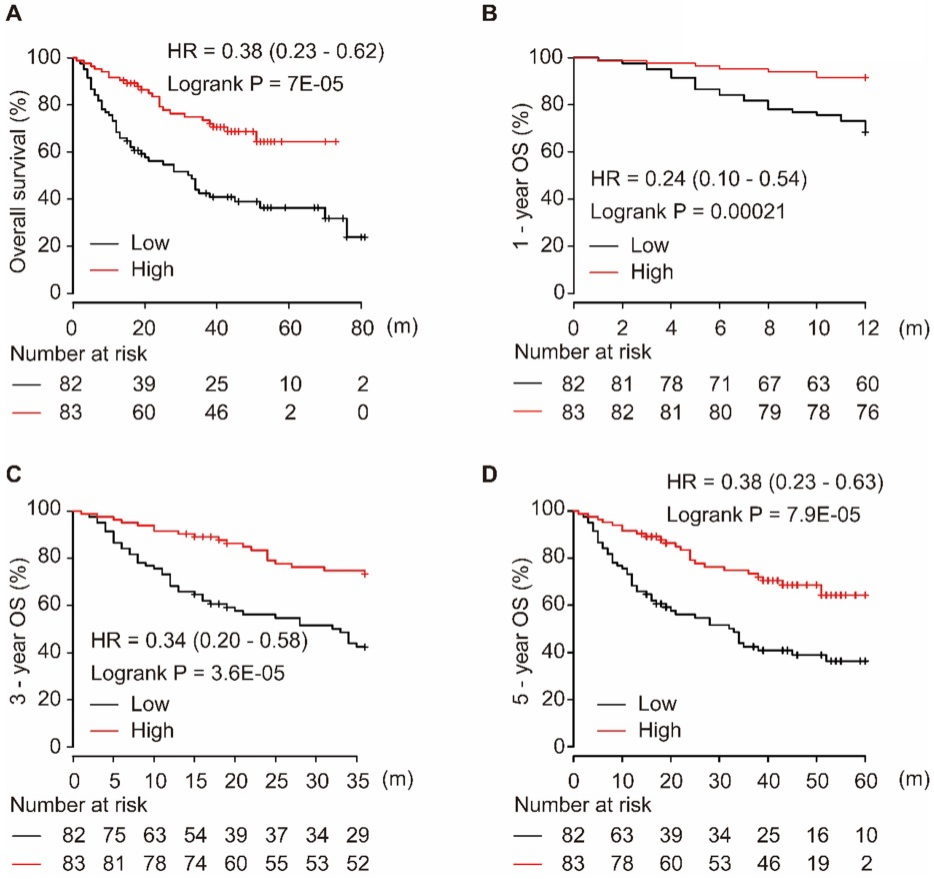
Low expression of miR-139-5p is associated with worse OS (A), 1-year (B), 3-year (C), and 5-year (D) OS in HCC patients.

Using the miRpower liver cancer miRNA database in Kaplan-Meier plotter, 166 HCC patients were included in the non-commercial spotted platform. As shown in Figure 1, downregulation of miR-139-5p was significantly associated with poor OS in HCC patients (*P* = 7E-05, Figure 1A). Similarly, a low level of miR-139-5p in tumors significantly correlated with the 1-year, 3-year, and 5-year OS in HCC patients (all *P* < 0.001, Figure 1B-D).

**Figure 2:**
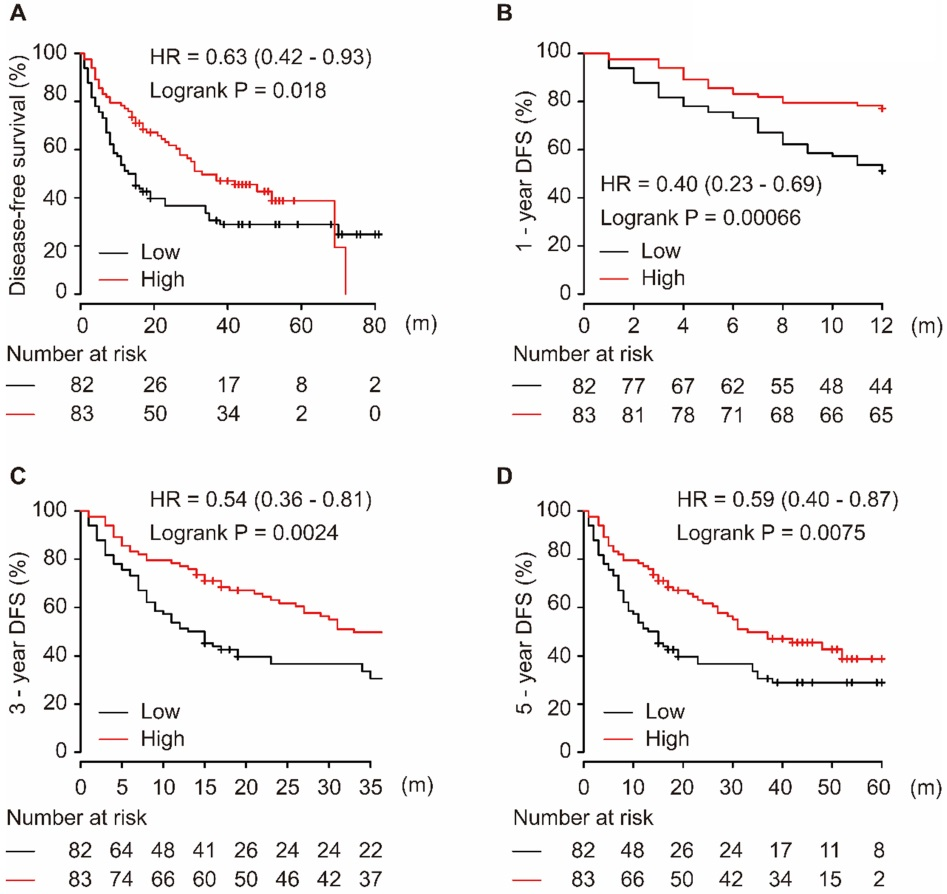
Low expression of miR-139-5p is associated with worse DFS (A), 1-year (B), 3-year (C), and 5-year (D) DFS in HCC patients.

As shown in Figure 2, the downregulation of miR-139-5p in tumor tissues was significantly associated with worse DFS in patients with HCC (*P* = 0.018, Figure 2A). Additionally, low expression of miR-139-5p significantly contributed to worse 1-year, 3-year, and 5-year DFS in HCC patients (all *P* < 0.01, Figure 2B-D).

### Target gene expression analysis

**Figure 3:**
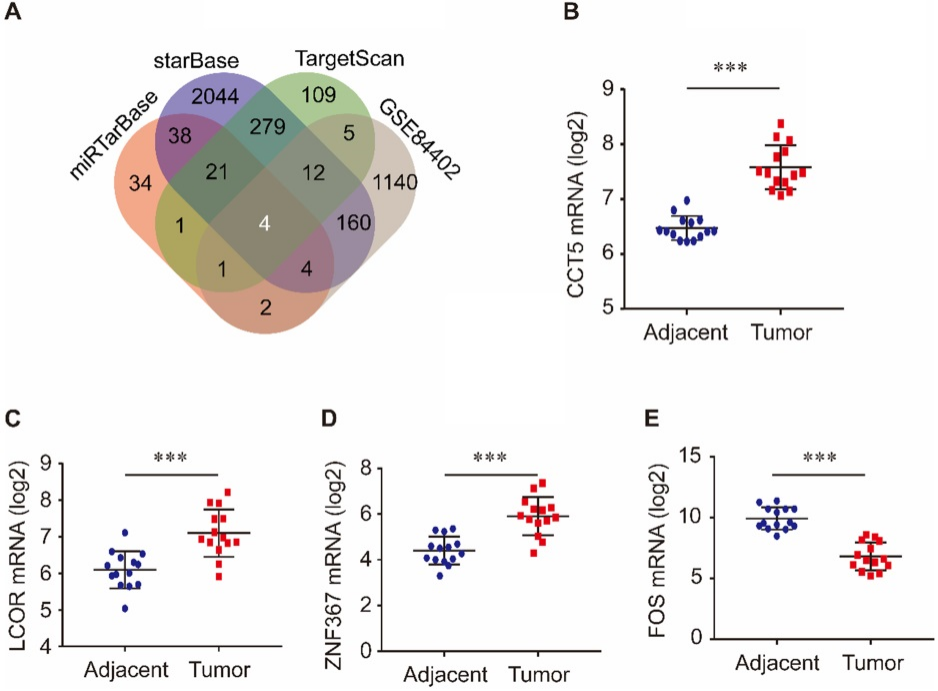
Target genes of miR-139-5p and its common genes with differential expression genes in GSE84402 (A); CCT5 (B), LCOR (C), ZNF367 (D) and FOS mRNA (E) expression in tumor and adjacent tissues in GSE84402.

Target genes of miR-139-5p were identified in the TargetScan, miRTarBase, and starBase databases. As shown in Figure 3A, 432 target genes were identified in TargetScan, 105 were identified in miRTarBase and 2562 were identified in starBase. Furthermore, we investigated the common genes between these target genes and the DEGs in the GSE84402 dataset. Four genes, CCT5, FOS, LCOR, and ZNF367, were identified. As shown in Figure 3, CCT5, LCOR, and ZNF367 were evidently overexpressed in tumor tissues compared with adjacent tissues (all *P* < 0.001, Figure 3B-D), while FOS was significantly downregulated in tumor tissues compared with non-tumor tissues of HCC patients (*P* < 0.001, Figure 3E).

### Association between CCT5 and overall survival in HCC

**Figure 4:**
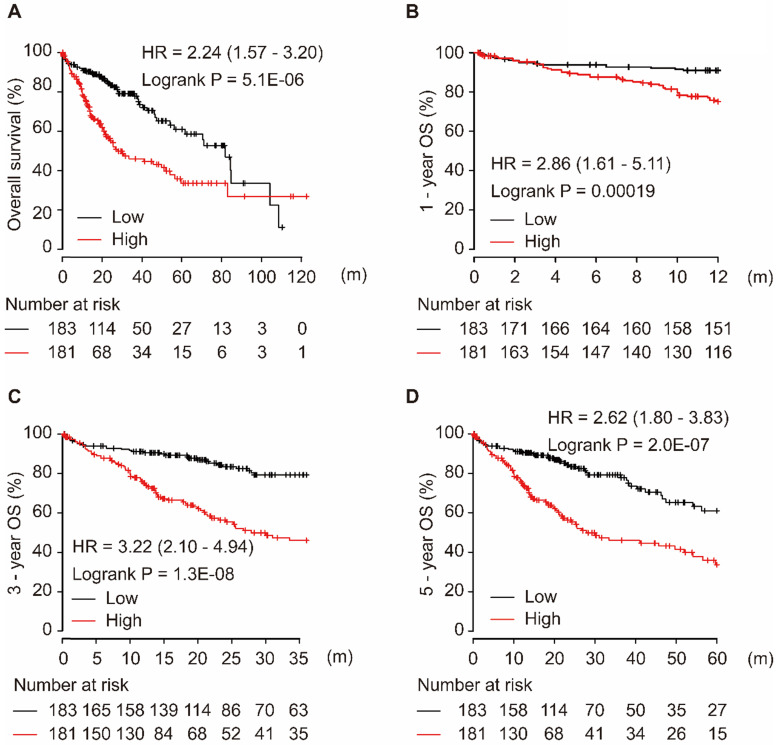
CCT5 mRNA overexpression is associated with worse OS (A), 1-year (B), 3-year (C), and 5-year (D) OS in HCC patients.

Among the four target genes (CCT5, FOS, LCOR, and ZNF367), only CCT5 showed significance for OS in HCC patients. As shown in Figure 4, CCT5 overexpression was significantly associated with worse OS in HCC patients (HR = 2.24, 95% CI = 1.57-3.20, *P* = 5.1E-06, Figure 4A). Moreover, a high level of CCT5 in tumor tissues was significantly associated with 1-year, 3-year, and 5-year OS in HCC patients (all HR > 2.0, *P* < 0.001, Figure 4B-D).

**Figure 5:**
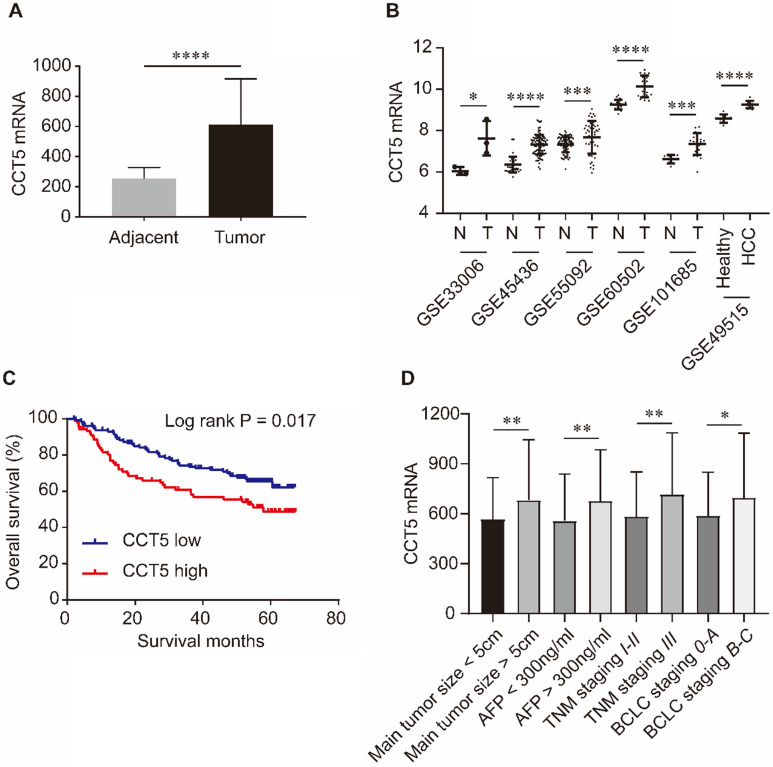
CCT5 mRNA expression and its associations with OS and clinico-pathological features in HCC patients. CCT5 mRNA was upregulated in tumor tissues than that in adjacent tissues in GSE14520, GSE33006, GSE45436, GSE55092, GSE60502 and GSE101685 (A, B); CCT5 mRNA was significantly increased in PBMCs of HCC patients than that in healthy individuals in GSE49515 (B); high CCT5 mRNA was associated with worse OS (C), CCT5 mRNA was upregulated in HCC patients with main tumor size > 5cm, AFP > 300 ng/ml BCLC stage B-C and/or TNM stage III (D).

As shown in Figure 5, CCT5 mRNA was significantly upregulated in tumor tissues compared with adjacent tissues in the GSE14520, GSE33006, GSE45436, GSE55092, GSE60502 and GSE101685 datasets (all *P* < 0.05, Figure 5A,B). In addition, CCT5 mRNA was also significantly increased in the PBMCs of HCC patients compared to those of healthy individuals in the GSE49515 dataset (*P* < 0.0001, Figure 5B). High expression of CCT5 mRNA in tumors was a risk factor for OS in HCC patients (log-rank P = 0.017, Figure 5B). Additionally, CCT5 mRNA was significantly overexpressed in HCC patients with alpha-fetoprotein (AFP) > 300 ng/ml, BCLC staging B-C, TNM staging III and main tumor size > 5 cm (Figure 5C-F, respectively; all *P* < 0.05).

### Correlation between miR-139-5p and CCT5

**Figure 6:**
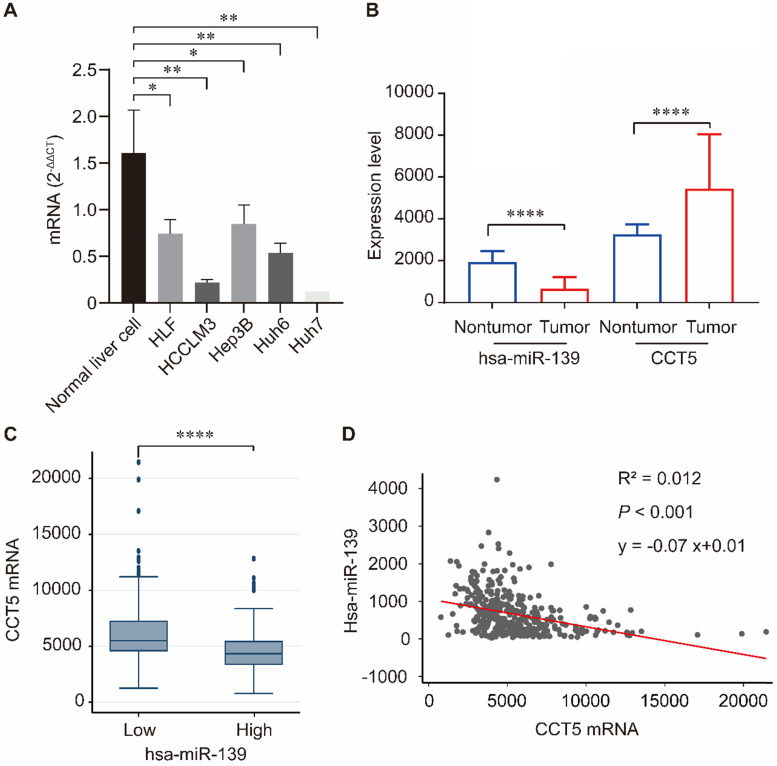
miR-139 was significantly downregulated in hepatoma cell lines compared to that in normal liver cells(A); miR-139 was also significantly downregulated in HCC tumors and CCT5 mRNA was significantly overexpressed in HCC tumors (B); CCT5 mRNA was significantly downregulated in miR-139 high samples (C); Negatively correlation was found between miR-139 and CCT5 mRNA in TCGA dataset (D).

Human miR-139-5p expression was significantly downregulated in hepatoma cell lines including HLF, HCCLM3, Hep3B, Huh6, and Huh7 compared to that in normal liver cells (all *P* < 0.05, Figure 6A). We investigated the correlation between miR-139 and CCT5 in the TCGA dataset. As summarized in Figure 6, miR-139 expression was significantly downregulated in HCC tumors (*P* < 0.0001, Figure 6B), and CCT5 mRNA was significantly overexpressed in HCC tumors (*P* < 0.0001, Figure 6B). In addition, CCT5 mRNA was significantly downregulated in miR-139 high group (*P* < 0.0001, Figure 6C). As expected, a negative correlation was found between miR-139 and CCT5 mRNA (*P* < 0.001, Figure 6D).

### Cox proportional hazard regression model

**Figure 7:**
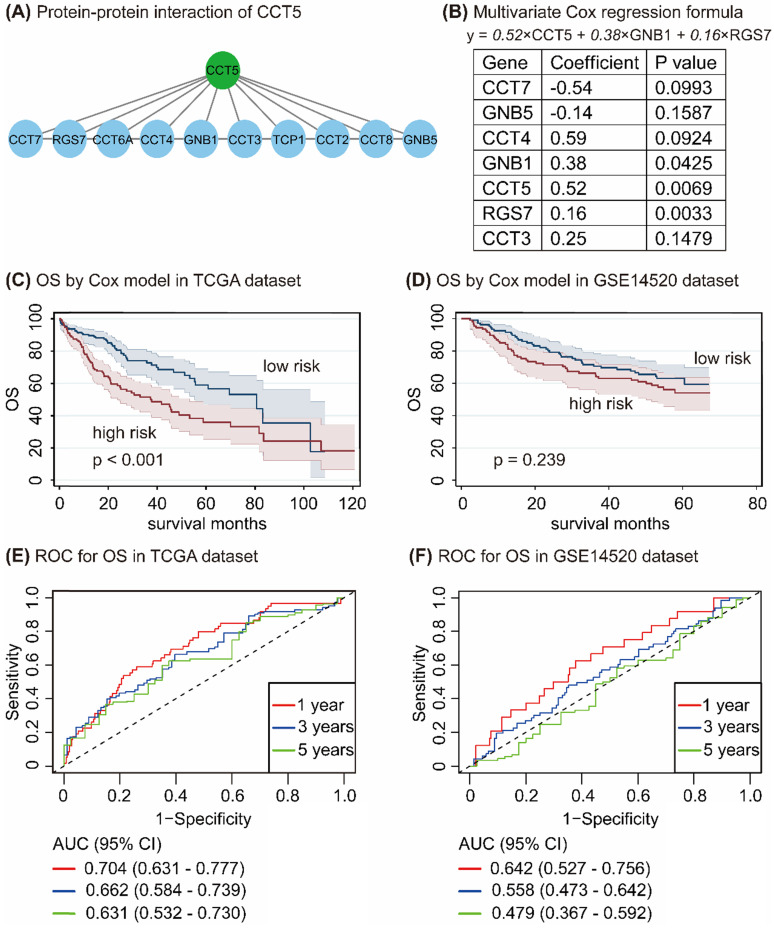
Protein-protein-interaction of CCT5 (A); Cox proportional hazard regression model of CCT5 interactive genes (B); Survival analysis based on Cox model grouped by risk scores in the TCGA dataset (C) and the GSE14520 dataset (D); and ROC curves of Cox model for OS in HCC patients in the TCGA dataset (E) and the GSE14520 dataset (F).

In the STRING database, 10 genes, namely, CCT7, RGS7, CCT6A, CCT4, GNB1, CCT3, TCP1, CCT2, CCT8, and GNB5, have interacted with CCT5 (Figure 7A). Univariate and multivariate Cox regression analyses in R program identified 7 genes, namely, CCT7, GNB5, CCT4, GNB1, CCT5, RGS7, and CCT3. CCT5, GNB1, and RGS7 were significantly associated with OS in HCC patients (all *P* < 0.05, Figure 7B), with the following formula: y = 0.52 × CCT5 + 0.38×GNB1 + 0.16 × RGS7 (Figure 7B). According to values obtained with the formula, HCC patients were divided into high-risk and low-risk groups in the TCGA and the GSE14520 datasets. As shown in Figure 7, HCC patients with high risk had poor OS compared with those of low-risk patients in the TCGA dataset (*P* < 0.001, Figure 7C), while no difference between this formula model and OS in HCC patients was found in the GSE14520 dataset (*P* = 0.239, Figure 7D). To determine the accuracy of this model for the prediction of the OS in HCC patients, the ROC curves with an area under the curve (AUC) in the TCGA and GSE14520 datasets were summarized in Figure 7E and Figure 7F, respectively.

## Discussion

Consistent with previous reports 32-34, our results revealed that the downregulation of miR-139-5p showed a significant association with worse survival in HCC patients. According to existing publications, miR-139-5p inhibits HCC progression by targeting several genes, such as ZEB1 and ZEB2 31, EZH2 52, SPOCK1 32, ETS1 33, XIST 53 and SLITRK4 34. MiR-139-5p was confirmed to negatively regulate ZEB1 and ZEB2 expression 31, which induces EMT and promotes the progression of malignant carcinoma 54, while overexpression of ZEB1 and ZEB2 abolishes the inhibitory effects of miR-139-5p on HCC cell migration and invasion 31. A previous report revealed that the zeste homolog 2 (EZH2) enhancer could promote HCC motility *in vitro* and pulmonary metastasis in a nude mouse model through epigenetically suppressing miR-139-5p by potent and extensive regulation of various signaling pathways involved in cell motility and metastasis 52. As an oncogene, SPOCK1 blocks apoptosis and promotes HCC metastasis 55. Overexpression of miR-139-5p suppressed HCC cell viability and invasion by targeting SPOCK1, promoted apoptosis, and inhibited tumor growth 32. A study by Hua et al. showed that miR-139-5p-induced aerobic glycolysis, proliferation, migration, and invasion were reversed by ETS1 overexpression, whereas ETS1 silencing induced the expression of miR-139-5p through posttranscriptional regulation 33. LncRNA X inactivate-specific transcript (XIST) induces the proliferation of HCC cells by promoting the cell cycle and protecting cells from apoptosis, and the mutual inhibitory action of XIST and miR-139-5p has also been investigated 53. Recently, SLITRK4 has been shown to be involved in the progression of HCC and to be targeted by miR-139-5p 34.

In addition to the target genes mentioned above, our analysis revealed that chaperonin-containing TCP1 subunit 5 (CCT5) might be a novel target gene of miR-139-5p linked to HCC survival and clinico-pathological features. CCT5 is a member of the chaperonin-containing TCP1 complex (CCT), also known as the TCP1 ring complex (TRiC). Its protein-folding activity is critical for cellular health. Misfolded proteins are associated with many human diseases, including cancer, making them potential therapeutic targets 56, 57. Highly expressed in cancer cell lines, CCT/TRiC is implicated in the folding of oncoproteins cyclin E, cyclin B, and p21(ras), which strongly indicates that it is involved in cell proliferation and tumorigenesis 58. CCT5 has mainly been investigated in neuropathology 59, 60. In HCC, CCT5 expression was upregulated in liver tumors, predicted shortened OS and DFS times. Gene enrichment analysis revealed that CCT5 was involved in the dysregulation of Myc target genes, hypoxia-inducible factor (HIF) target genes and the cell cycle, especially the G1/S transition, cell cycle arrest and apoptosis 61, 62. Additionally, CCT5 mutations might be associated with the accumulation of cytotoxic protein aggregates with tissue destruction and mitochondrial damage 63. In breast cancer, overexpression of CCT5 correlated with tumor purity and immune infiltration levels and was associated with poor survival rates 64. CCT5 mRNA was significantly upregulated in p53-mutated tumors and associated with a low response rate to docetaxel in breast cancer. Treatment of MCF-7 cells with CCT5-specific siRNA resulted in a significant enhancement of apoptosis induced by docetaxel 65. In non-small cell lung cancer (NSCLC), CCT5 was expressed at higher levels in tumor tissues and induced an autoantibody response in NSCLC sera 66. In addition, CCT5 was also overexpressed in sinonasal adenocarcinoma 67, esophageal squamous cell carcinoma 68 and multidrug-resistant gastric carcinoma cells 69. We suggest mechanistic insights into the role of CCT5 in tumorigenesis and tumor progression in the HCC population that should be considered in the future.

There are some limitations to our study. First, even though the suppressive impact of miR-139-5p on CCT5 was addressed in this study, our analysis did not involve experimental assays to verify the reciprocal repression between miR-139-5p and CCT5. Second, no experiments were performed to address the effects of the miR-139-5p/CCT5 axis on HCC cellular functions; Third, follow-up data of HCC patients from our own dataset were not available; And, the extension of Cox model in our research was relatively unfavorable in the GSE14520, further investigations are needed. However, according to our integrated analysis, we cautiously assumed that the miR-139-5p/CCT5 axis might be a novel mechanism in the progression of HCC. Since the hypothesis was based on a bioinformatic research, further investigations regarding the associations between miR-139-5p and CCT5 should be considered.
